# Enantioselective [4+1] Annulation of Benzamide with Cyclopropene via Chiral Cp^X^Co(III) Catalysis: A DFT Perspective

**DOI:** 10.1021/acsomega.6c01239

**Published:** 2026-09-10

**Authors:** Bat-El Oded, Haya Kornweitz

**Affiliations:** Department of Chemical Sciences, 42732 Ariel University, Ariel 4070000, Israel

## Abstract

Asymmetric C–H bond functionalization employing Cp^X^M­(III) has gained significant attention in organic synthesis transformations. In this work, the first comprehensive DFT investigation has been performed to gain a better understanding of the origin of enantioselectivity and regioselectivity of Co-catalyzed asymmetric [4+1] annulation of *N*-chlorobenzamide with cyclopropenes. Our DFT calculations indicate that, although the microkinetic model failed to predict the *trans-endo/cis-endo* product ratio, the ratio is within the typical intrinsic error margins of DFT calculations. This thorough DFT analysis is a key to advancing the rational design of enantioselective C–H functionalization transformations with chiral Cp^X^M­(III) catalysts.

## Graphical abstract

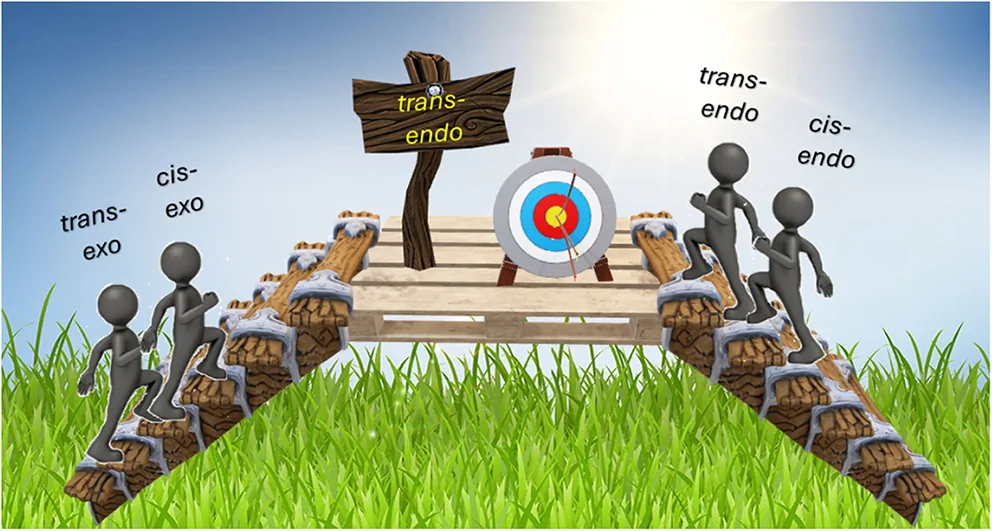

## Introduction

Chiral carbon–carbon (C–C) and carbon-heteroatom (C–X) bonds are very common in natural products and biologically active molecules. Among the methodologies in use, direct asymmetric functionalization of C–H bond via transition metals represents one of the most powerful approaches due to atom economy.^1–3^ Over the past decades, a wide range of transition-metal catalysts have been explored for C–H functionalization reactions. Notably, Cp*M­(III) catalysts (Cp* = 1,2,3,4,5-pentamethylcyclopentadiene, M=Co, Rh, Ir), featuring a pentamethylcyclopentadienyl ligand, have emerged as top performers, offering superior reactivity, excellent functional group tolerance, and outstanding robustness.^2^ In addition, a myriad of chiral Cp (Cp^X^) ligands are accessible by the installation of chiral backbones on the Cp ring.^4–6^ For example, there are Cp ligands bearing three substituents that may create different steric as well as electronic environments around the metal center, altering the factors controlling enantioselectivity.^7^ The first rational design of chiral Cp^X^ ligands was proposed by Cramer in 2012.^8^ In this model, the enantioinduction is rationalized by steric repulsion with a “side wall” moiety that controls the orientation of the metallacycle, whereas a “back wall” moiety forces the substrate to approach from the opposite side.^2^

In 2025, the Cramer group reported a [4+1] annulation of *N*-chlorobenzamide with cyclopropenes using a chiral Cp^X^Co­(III) catalyst to give chiral isoindolinones, a common structure in biologically active molecules (Scheme 1).^9^ This reaction is carried out in a 1:1 mixture of 2,2,2-trifluoroethanol (TFE) and 1,2-dichloroethane (DCE) at 23 °C for 36 h using a chiral cobalt catalyst with a trisubstituted BINOL-Cp. Additionally, high enantiomeric excess and selectivity that favor the (*E*)-olefin geometry were achieved across a broad range of substrates.

**Scheme 1:**
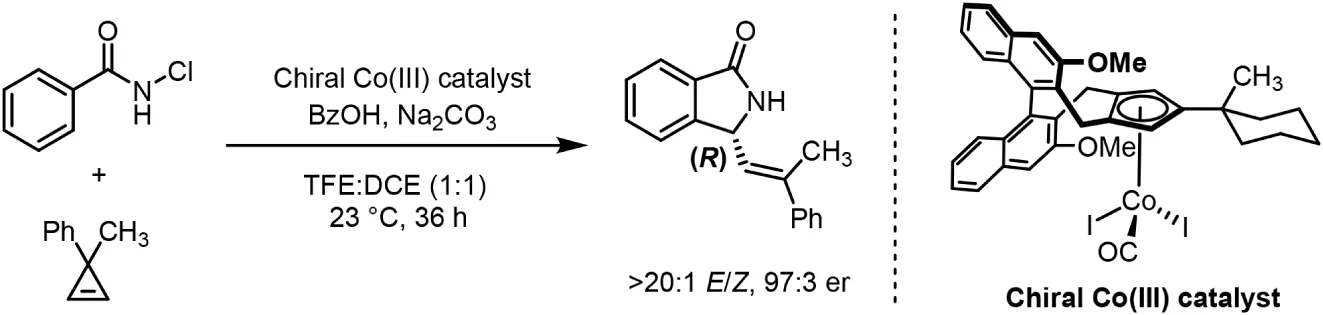
Co­(III)-Catalyzed Enantioselective [4+1] Annulation of *N*-Chlorobenzamide with Cyclopropene, Copied from ref 9. (CC-BY 4.0)^9^

To gain mechanistic insight into the reaction profile, the Cramer group performed DFT calculations to explore possible pathways with Cp* and 3,3-dimethylcyclopropene (Scheme 2).^9^ First, migratory insertion of cyclopropene into five-membered cobaltacycle **Int1** can afford seven-membered aza-cobaltacycle **Int3** through **Int2**. In the next step, ring opening leads to six-membered aza-cobaltacycle **Int4**. Then, Co­(V)-nitrenoid (**Int5** ^**C**^) is formed, which readily undergoes nitrene insertion (**Int6**) and with the Co–N bond and protonation to give the [4+1] annulation product. Induced intersystem crossing to the triplet state of species **Int4** led to the final annulation product. It was proposed that the ring opening of cyclopropane leads to the thermodynamically favorable (*E*)-product of intermediate **Int4**, while the pathway leading to (*Z*) olefin geometry is less favorable due to possible steric hindrance between the phenyl group of cyclopropene and the binaphthyl backbone of the chiral Co catalyst. However, DFT calculations have been performed on an achiral Cp* ligand and dimethylcyclopropene.^9^ In this case, enantioselectivity and regioselectivity are omitted, leaving the mechanism of these selectivities unexplored.

**Scheme 2:**
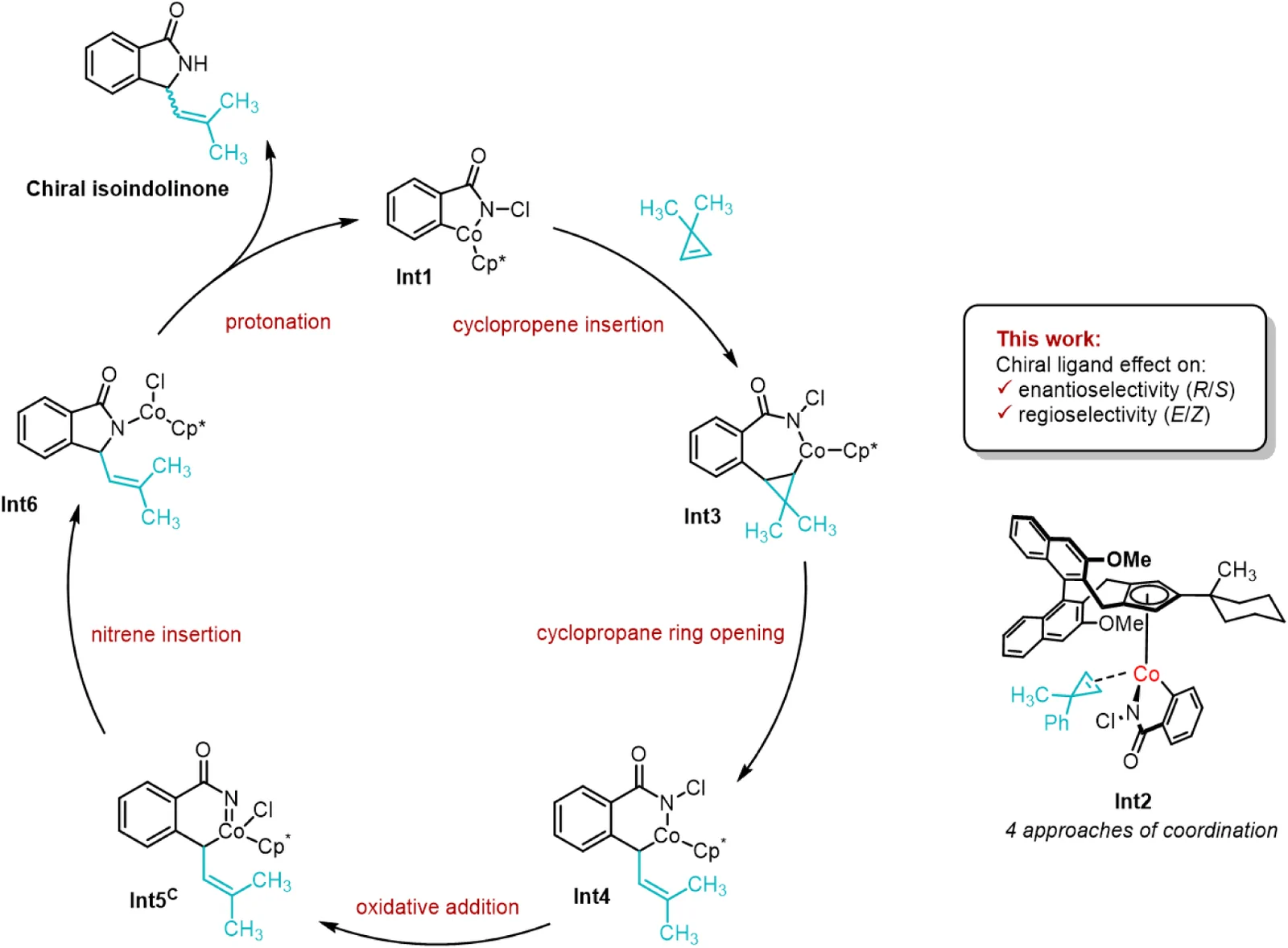
Cramer’s DFT Calculated Mechanism of [4+1] Annulation of Benzamide with Cyclopropene, Data from ref 9. (CC-BY 4.0)^9^

While density functional theory (DFT) has proven invaluable for elucidating enantioselectivity and mechanistic pathways in transition metal-catalyzed reactions such as Rh­(III),^10,11^ a comprehensive computational investigation addressing enantioselectivity and regioselectivity in C–H bond functionalization reactions mediated by chiral Cp^X^Co­(III) is still limited. In this work, we conducted the first computational study for a better understanding of enantioselectivity and regioselectivity of C–H bond activation of benzamide using a chiral Cp^X^Co­(III) catalyst (Scheme 1). We aim to examine the factors governing both regio- and enantioselectivity in the [4+1] annulation reaction and elucidate the preferential formation of the *R*-enantiomer with *E*-selectivity. A profound computational understanding of how ligand architecture and substrate orientation influence stereochemical outcomes is essential for providing guidelines for the rational design of new transformations and catalysts.

## Computational Details

All calculations were carried out using Gaussian 16.^12^ Geometry optimizations were performed in both the singlet and triplet (unrestricted) states with the M06 density functional.^13^ The 6–311G^**^ basis set was applied for all atoms^14^ except Co, which was described by the SDD basis set,^15^ with dispersion correction (GD3).^16^ Vibrational frequency analysis was performed to calculate the zero-point energy considered in calculating Gibbs free energies and to validate the nature of the calculated species, zero imaginary frequencies for a stationary point, and one imaginary frequency for a transition state (TS). Solvation energy corrections were calculated using the SMD model with trifluoroethanol (TFE) and 1,2-dichloroethane (DCE) as solvents (Schemes S1 and S2).^17^ Since the reaction was carried out in a 1:1 solvent mixture of DCE:TFE, the calculations were performed separately in both solvents, and the average energy value was used^18^ (Table S1). The trends of the results are not affected by changing the solvent or taking an average. TS was found through a scan calculation followed by TS (opt = ts, calcfc). The imaginary frequency was monitored to validate whether it leads to the desired product.

The superscript C is for Cramer, S is for singlet state, and T is for triplet state.

An extensive automated conformational search (CREST)^19^ was used to determine the best conformations of **Int2**, **Int3** ^**S**^, and **TS1**.

NCI calculations were performed using NCIWEB with a cutoff of 0.3 a.u.^20^

## Results and Discussion

The process starts with the coordination of cyclopropene to **Int1**. The triplet state of **Int1** is more stable than the singlet state by 6.0 kcal mol^–1^ with K_eq_ = 4 × 10^–5^, requiring spin-crossover event to the singlet state, however, the cyclopropene insertion is possible only with the singlet **Int1**, as the concentration of singlet **Int1** is very low, the reaction is very slow, but as the process is highly exergonic, it drives the equilibrium towards the generation of more singlet **Int1**. The coordination of cyclopropene to **Int1** (Scheme 3) in the singlet state forms a π complex (**Int2**, Figure 1) in a slightly endergonic reaction (ΔG° = 0.7, 1.4, 6.2, and 6.7 kcal mol^–1^ for *cis*-*endo* (*R*, *Z*), *trans*-*endo* (*R*, *E*), *cis*-*exo* (*S*, *Z*) and *trans*-*exo* (*S*, *E*), respectively). The formation of the *endo*-diastereomers is preferred thermodynamically. Next, we confirmed that the facial selectivity of the coordination of cyclopropene following migratory insertion is the diastereo-determining step,^2^ computing the barriers in the forward and reverse directions for each of the four conformations. For both diastereofaces of the cyclopropene, two diastereomers exist in which the disubstituted carbon is oriented towards (*endo*) or away (*exo*) from the benzamide. The C–C bond formation step indicates a facile migratory insertion with barriers (**TS1**) of 4.8 and 5.7 kcal mol^–1^ for *cis*-*endo* (*R*, *Z*) and *trans*-*endo* (*R*, *E*), respectively (Scheme 3), while for the *exo* approach, the barriers are 12.5 and 37.4 kcal mol^–1^ for the *cis*-*exo* (*S*, *Z*) and *trans*-*exo* (*S*, *E*), respectively, to form **Int3** ^**S**^. The calculations show that the step of forming **Int3** ^**S**^ is exergonic (ΔG° = −18.1, −20.3, −10.7 and −13.8 kcal mol^–1^ for *cis*-*endo* (*R*, *Z*), *trans*-*endo* (*R*, *E*), *cis*-*exo* (*S*, *Z*) and *trans*-*exo* (*S*, *E*), respectively), causing the barriers in the reverse direction to be prohibitively large (22.9 and 26.0 kcal mol^–1^ for *cis*-*endo* (*R*, *Z*) and *trans*-*endo* (*R*, *E*), respectively), significantly reducing the plausibility of the reverse reaction, determining the diastereoselectivity, with thermodynamic and kinetic preference for the *endo* diastereomers. The high transition-state barriers and the lower exergonicity associated with the *exo* approach effectively rule out its formation relative to the *endo* pathway.

**Figure 1:**
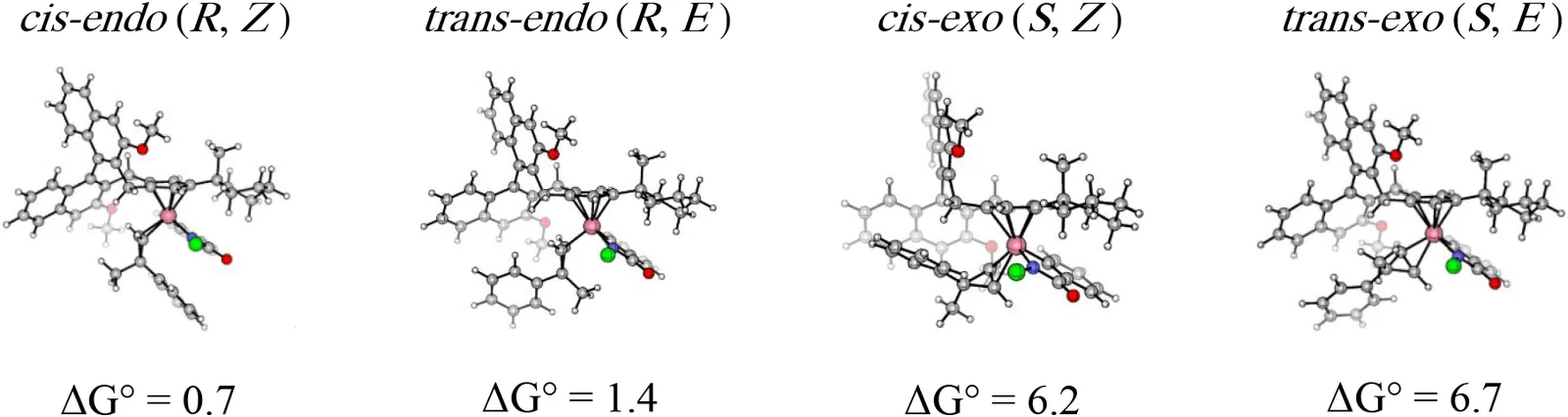
Plausible structures of the π complex **Int2**, formed by four approaches of cyclopropene and **Int1**, ΔG° values for this reaction in kcal mol^–1^.

**Scheme 3:**
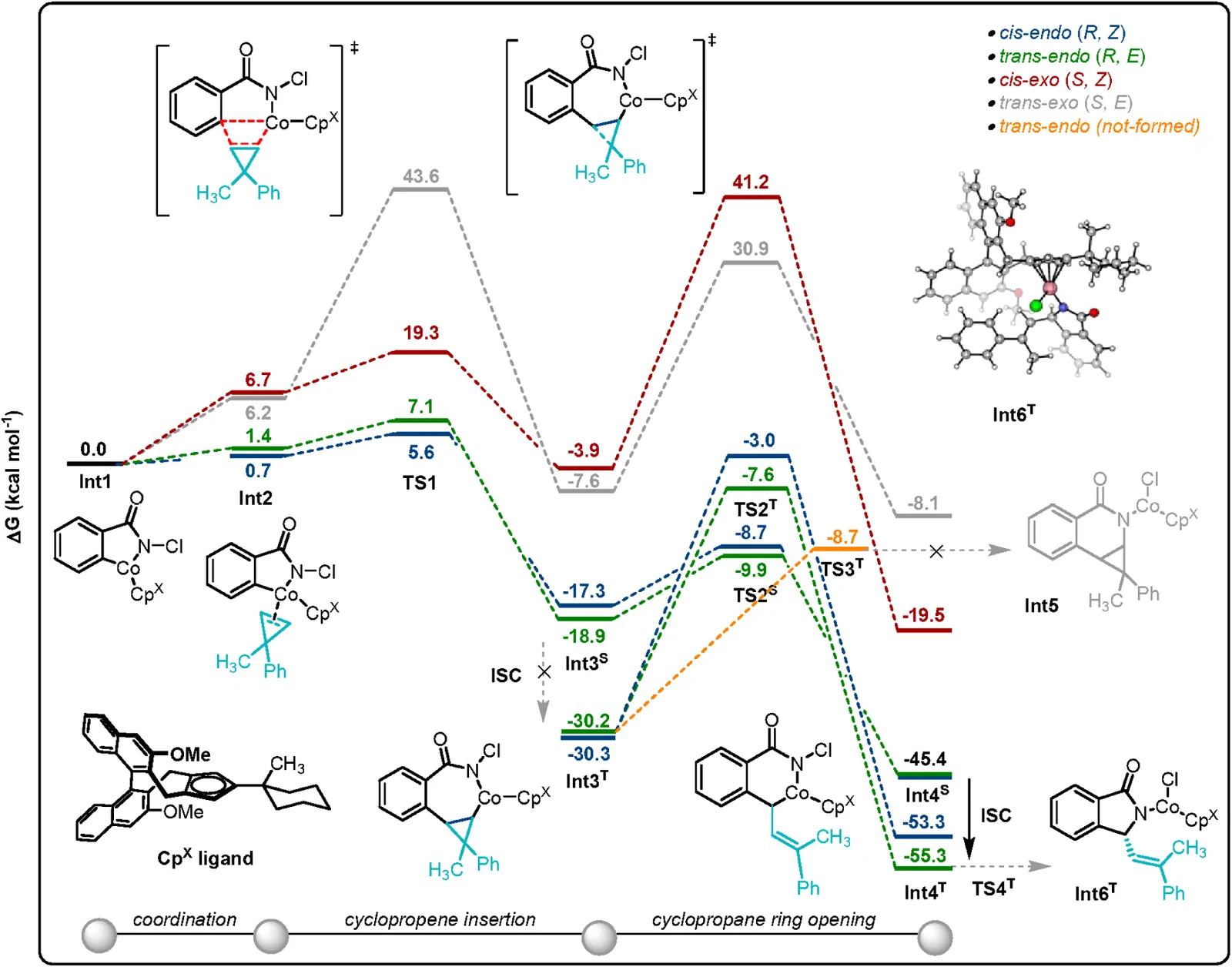
Energy Profile Comparison between the Singlet and Triplet States for *Cis-Endo* (*R*, *Z*), *Trans-Endo* (*R*, *E*), *Cis*-*Exo* (*S*, *Z*), and *Trans*-*Exo* (*S*, *E*) Approaches^a^

a DFT calculations were performed at the M06/SDD-6-311G**/SMD­(TFE, DCE) level of theory (blue: *cis*-*endo* (*R*, *Z*), green: *trans*-*endo* (*R*, *E*), red: *cis*-*exo* (*S*, *Z*), grey: *trans*-*exo* (*S*, *E*), orange: not formed, ΔG° and ΔG^‡^ in kcal mol^–1^. This scheme follows the scheme in ref 9 (CC-BY 4.0)^9^.

In the cyclopropane ring-opening step, a six-membered aza-cobaltacycle **Int4** ^**S**^ (ΔG° = −28.1, −26.5, −15.5 and −0.5 kcal mol^–1^ for *cis*-*endo* (*R*, *Z*), *trans*-*endo* (*R*, *E*), *cis*-*exo* (*S*, *Z*), and *trans*-*exo* (*S*, *E*), respectively) is formed via **TS2** ^**S**^ (ΔG^‡^ = 8.6, 8.9, 45.2, and 38.6 kcal mol^–1^ for *cis*-*endo* (*R*, *Z*), *trans*-*endo* (*R*, *E*), *cis*-*exo* (*S*, *Z*) and *trans*-*exo* (*S*, *E*), respectively). The barriers for the *exo* are extremely high, while the barriers for the *endo* are moderate. Based on the lower transition-state barriers, it is anticipated that the *trans*-*endo* (*R*, *E*) and *cis*-*endo* (*R*, *Z*) products are the major species formed, excluding the formation of the *exo* products.

The predominant steps are‡

1a $$\begin{array}{l} {{\mathrm{Int}2}^{S}}_{cis‐endo\left(R,Z\right)}\to {{\mathrm{Int}3}^{S}}_{cis‐endo\left(R,Z\right)}\\ {\Delta G}^{{}^{\circ}}=‐18.1\;{\mathrm{kcal mol}}^{‐1},\Delta G^{‡}\left(\mathrm{TS1}\right)=4.8\;{\mathrm{kcal mol}}^{‐1},{k_{2\to 3}}^{\mathrm{cis‐endo}(R,Z)}=3.0\times 10^{8} \end{array}$$

1b $$\begin{array}{l} {{\mathrm{Int}2}^{S}}_{trans‐endo\left(R,E\right)}\to {{\mathrm{Int}3}^{S}}_{trans‐endo\left(R,E\right)}\\ {\Delta G}^{{}^{\circ}}=-20.3\;{\mathrm{kcal}\;\mathrm{mol}}^{\mathrm{-1}},\Delta G^{‡}\left(\mathrm{TS1}\right)=5.7\;{\mathrm{kcal}\;\mathrm{mol}}^{\mathrm{-1}},{k_{2\to 3}}^{trans‐endo(R,E)}=6.5\times 10^{7} \end{array}$$

2a $$\begin{array}{l} {{\mathrm{Int}3}^{S}}_{cis‐endo\left(R,Z\right)}\to {{\mathrm{Int}4}^{S}}_{cis‐endo\left(R,Z\right)}\\ \Delta G^{{}^{\circ}}=\mathrm{-28.1}\;{\mathrm{kcal}\;\mathrm{mol}}^{-1},\Delta G^{‡}\left(\mathrm{TS2}\right)=8.6\;{\mathrm{kcal}\;\mathrm{mol}}^{\mathrm{-1}},{k_{3\to 4}}^{cis‐endo(R,Z)}=4.9\times 10^{5} \end{array}$$

2b $$\begin{array}{l} {{\mathrm{Int}3}^{S}}_{trans‐endo\left(R,E\right)}\to {{\mathrm{Int}4}^{S}}_{trans‐endo\left(R,E\right)}\\ \Delta G^{{}^{\circ}}=\mathrm{-26.5}\;{\mathrm{kcal}\;\mathrm{mol}}^{\mathrm{-1}},\Delta G^{‡}\left(\mathrm{TS2}\right)=8.9\;{\mathrm{kcal}\;\mathrm{mol}}^{\mathrm{-1}},{k_{3\to 4}}^{trans‐endo(R,E)}=2.9\times 10^{5} \end{array}$$

Rate constants were calculated using the equation: $k=10^{12}\times e^{-\Delta G^{‡}/RT}$ , R is the gas constant (1.987 cal mol^–1^ K^–1^), and T = 298 K.

The full set of forward and backward rate constants is summarized below:

$${k_{2\to 3}}^{cis‐endo}=3.0\times 10^{8},{k_{3\to 2}}^{cis‐endo}=1.6\times 10^{-5}$$

$${k_{2\to 3}}^{trans‐endo}=6.5\times 10^{7},{k_{3\to 2}}^{trans‐endo}=8.5\times 10^{-8}$$

$${k_{3\to 4}}^{cis‐endo}=4.9\times 10^{5},{k_{4\to 3}}^{cis‐endo}=1.2\times 10^{-15}$$

$${k_{3\to 4}}^{trans‐endo}=2.9\times 10^{5},{k_{4\to 3}}^{trans‐endo}=1.1\times 10^{-14}$$

Applying the microkinetic model and assuming a steady-state approximation for the [**Int3** ^**S**^] intermediate, yields the following net rate expression:

$$\frac{d\left[Int3^{S}\right]}{dt}=k_{2\to 3}\left[Int2^{S}\right]-k_{3\to 2}[Int3^{S}]-k_{3\to 4}[Int3^{S}]+k_{4\to 3}[Int4^{S}]=0$$

Because all intermediate concentrations share the same order of magnitude, the backward rate constants

$${k_{3\to 2}}^{cis‐endo}=1.6\times 10^{-5}$$

$${k_{3\to 2}}^{trans‐endo}=8.5\times 10^{-8}$$

$${k_{4\to 3}}^{cis‐endo}=1.2\times 10^{-15}$$

$${k_{4\to 3}}^{trans‐endo}=1.1\times 10^{-14}$$

are highly negligible relative to the forward rate constants

$${k_{2\to 3}}^{cis‐endo}=3.0\times 10^{8}$$

$${k_{2\to 3}}^{trans‐endo}=6.5\times 10^{7}$$

$${k_{3\to 4}}^{cis‐endo}=4.9\times 10^{5}$$

$${k_{3\to 4}}^{trans‐endo}=2.9\times 10^{5}$$.

Neglecting these minor reverse terms simplifies the steady-state equation to

$$\frac{d\left[Int3^{S}\right]}{dt}=k_{2\to 3}\left[Int2^{S}]-k_{3\to 4}[Int3^{S}\right]=0$$

Given that the initial coordination of cyclopropane to form **Int2** ^**S**^ is barrierless, the *cis-endo* and *trans-endo* conformers are generated at identical rates, resulting in equal starting concentrations. The ratio between the concentrations of **Int3** ^**S**^ and **Int2** ^**S**^ for *trans-endo*/*cis-endo* is

$$\frac{\frac{{[{Int3}^{S}]}^{trans‐endo}}{{[{Int2}^{S}]}^{trans‐endo}}}{\frac{{[{Int3}^{S}]}^{cis‐endo}}{{[{Int2}^{S}]}^{cis‐endo}}}=\frac{{[{Int3}^{S}]}^{trans‐endo}}{{[{Int3}^{S}]}^{cis‐endo}}=\frac{\frac{{k_{2\to 3}}^{trans‐endo}}{{k_{3\to 4}}^{trans‐endo}}}{\frac{{k_{2\to 3}}^{cis‐endo}}{{k_{3\to 4}}^{cis‐endo}}}=\frac{\frac{6.5\times 10^{7}}{2.9\times 10^{5}}}{\frac{3.0\times 10^{8}}{4.9\times 10^{5}}}=0.36$$

The $\frac{{[Int3^{S}]}^{trans‐endo}}{{[Int3^{S}]}^{cis‐endo}}$ ratio is supposed to predict the ratio of the *trans*-*endo* to *cis*-*endo* products. DFT calculation is not (even qualitatively) consistent with the experimental results; the DFT predicts that the *cis-endo* pathway to give the *Z*-isomer is slightly favored, but the *E*-isomer is experimentally favored. The experimental value is two orders of magnitude higher than the calculated value (>20:1 *E*/*Z*).^9^ This discrepancy corresponds to an energy variation of only 2.7 kcal mol^–1^ at 298 K, which falls well within the typical intrinsic error margins of DFT calculations.

The structures of the transition states and intermediates are shown in Figure 2 (Figure S1 for *exo* diastereomers).

**Figure 2:**
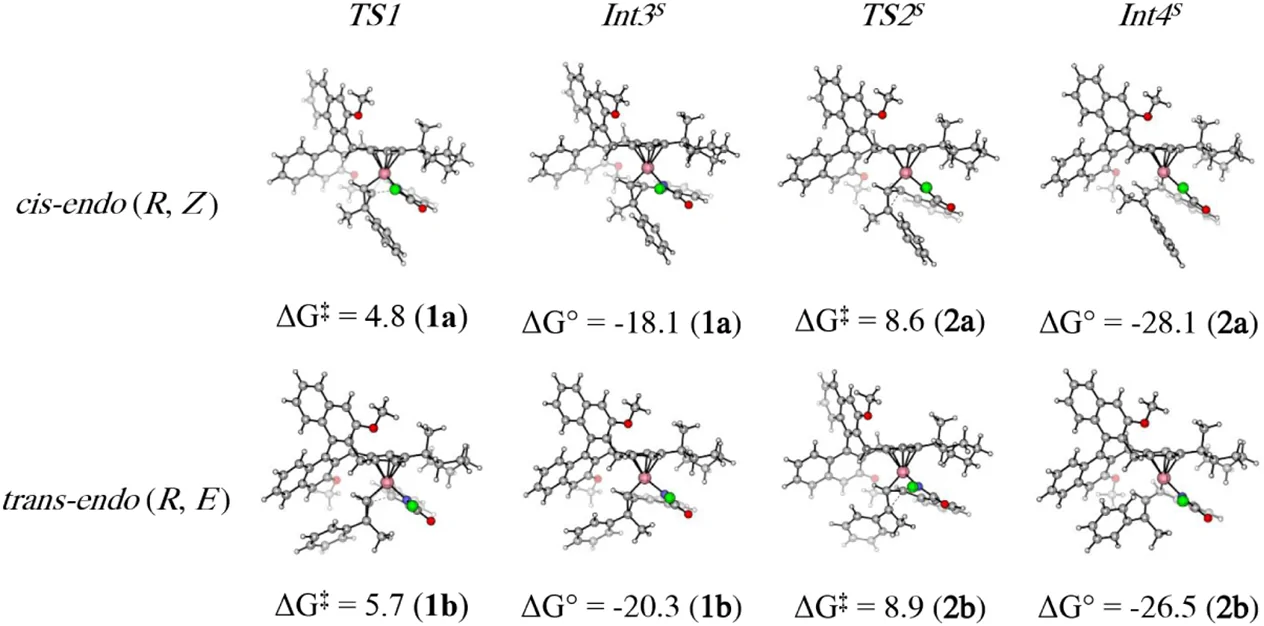
Optimized intermediates and respective transition states in the singlet state, ΔG° and ΔG^‡^ values for each reaction in kcal mol^–1^.

The nonbonding interactions of all four diastereomeric **TS1** (*cis*-*endo* (*R*, *Z*), *trans*-*endo* (*R*, *E*), *cis*-*exo* (*S*, *Z*), and *trans*-*exo* (*S*, *E*)) are evident from the visualization of the Non-Covalent Interaction isosurfaces (Figure 3). In all TS structures, there is a weak π interaction between the Cp^X^ ligand and the phenyl group of the cyclopropene (blue arrow). However, in the preferred **TS1** structures (*cis*-*endo* (*R*, *Z*) and *trans*-*endo* (*R*, *E*)) the interaction of the cyclopropane hydrogens and the ligand does not display characteristics of an unfavorable steric interaction, and the isosurface suggests weak π interactions. Additionally, both phenyl and methyl groups show attractive interaction with benzamide (black arrow) in the *cis*-*endo* (*R*, *Z*) and *trans*-*endo* (*R*, *E*) approaches, respectively. However, in the two TS of the unfavorable structures (*cis*-*exo* (*S*, *Z*) and *trans*-*exo* (*S*, *E*)), the hydrogens are oriented close to the benzamide, producing unavoidable clashes (black arrow), raising both TS barriers.

**Figure 3:**
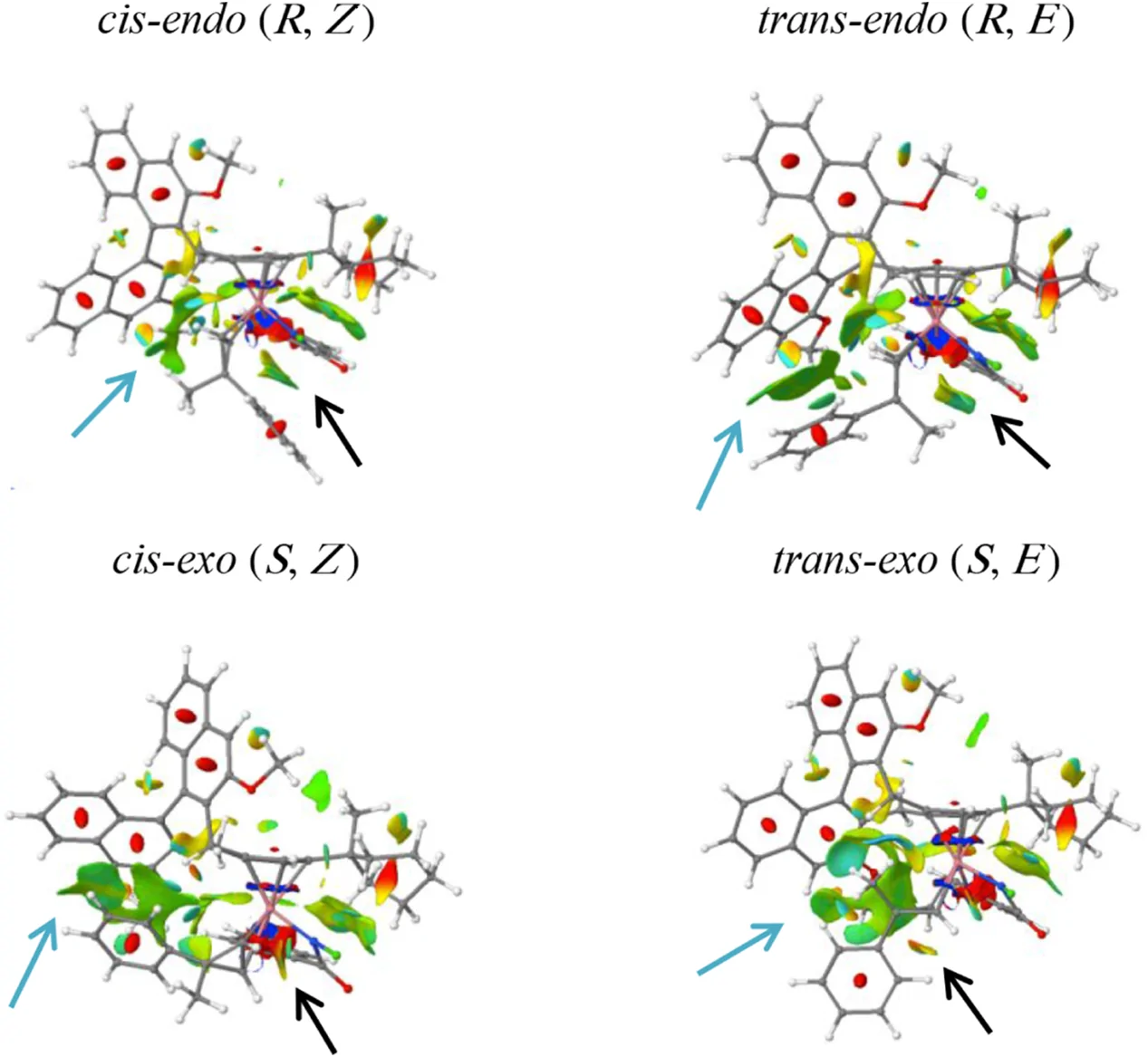
Non-covalent interaction (NCI) isosurface for **TS1** calculated by NCIWEB. Green – weak π interactions, red – steric repulsions.

Cramer *et al.* reported that **Int4** (Scheme 2) undergoes an ISC process to reach the final product.^9^ However, in a similar study, investigating Cp^X^Co­(III)­catalyzed annulation of *N*-chlorobenzamide with styrene, Li *et al.* reported that a similar species to **Int3** ^**S**^ undergoes the ISC process.^7^ Important to note, in their study, they explored the mechanism with a chiral Cp ligand. Thus, we decided to examine the plausibility of ISC step for the *cis*-*endo* (*R*, *Z*) and *trans*-*endo* (*R*, *E*) conformations of **Int3** ^**S**^. The calculations showed that **Int3** is more stable in its triplet state (**Int3** ^**T**^) than the singlet state by 13.0 and 11.3 kcal mol^–1^ for *cis*-*endo* (*R*, *Z*) and *trans*-*endo* (*R*, *E*), respectively (Scheme 3). Further calculations revealed that the transition state barrier for the cyclopropane ring opening (**TS2** ^**T**^) is 27.3 and 22.6 kcal mol^–1^ for *cis*-*endo* (*R*, *Z*) and *trans*-*endo* (*R*, *E*), respectively, while the barriers for the singlet-state **TS2** ^**S**^ are significantly lower (8.6 and 8.9 kcal mol^–1^ for *cis*-*endo* (*R*, *Z*) and *trans*-*endo* (*R*, *E*), respectively, (Scheme 3)). Also, **Int4** is more stable in the triplet state (**Int4** ^**T**^) then in its singlet state for both *cis*-*endo* (*R*, *Z*) and *trans*-*endo* (*R*, *E*) isomers (ΔG° = −23.0 and −25.1 kcal mol^–1^ for *cis*-*endo* (*R*, *Z*) and *trans*-*endo* (*R*, *E*), respectively). Based on these results, the cyclopropane ring-opening step is expected to proceed preferentially through the singlet pathway, since the transition state barriers are lower than those of the triplet route and the ISC is expected to occur at **Int4** ^**S**^. The structures are shown in Figure 4.

**Figure 4:**
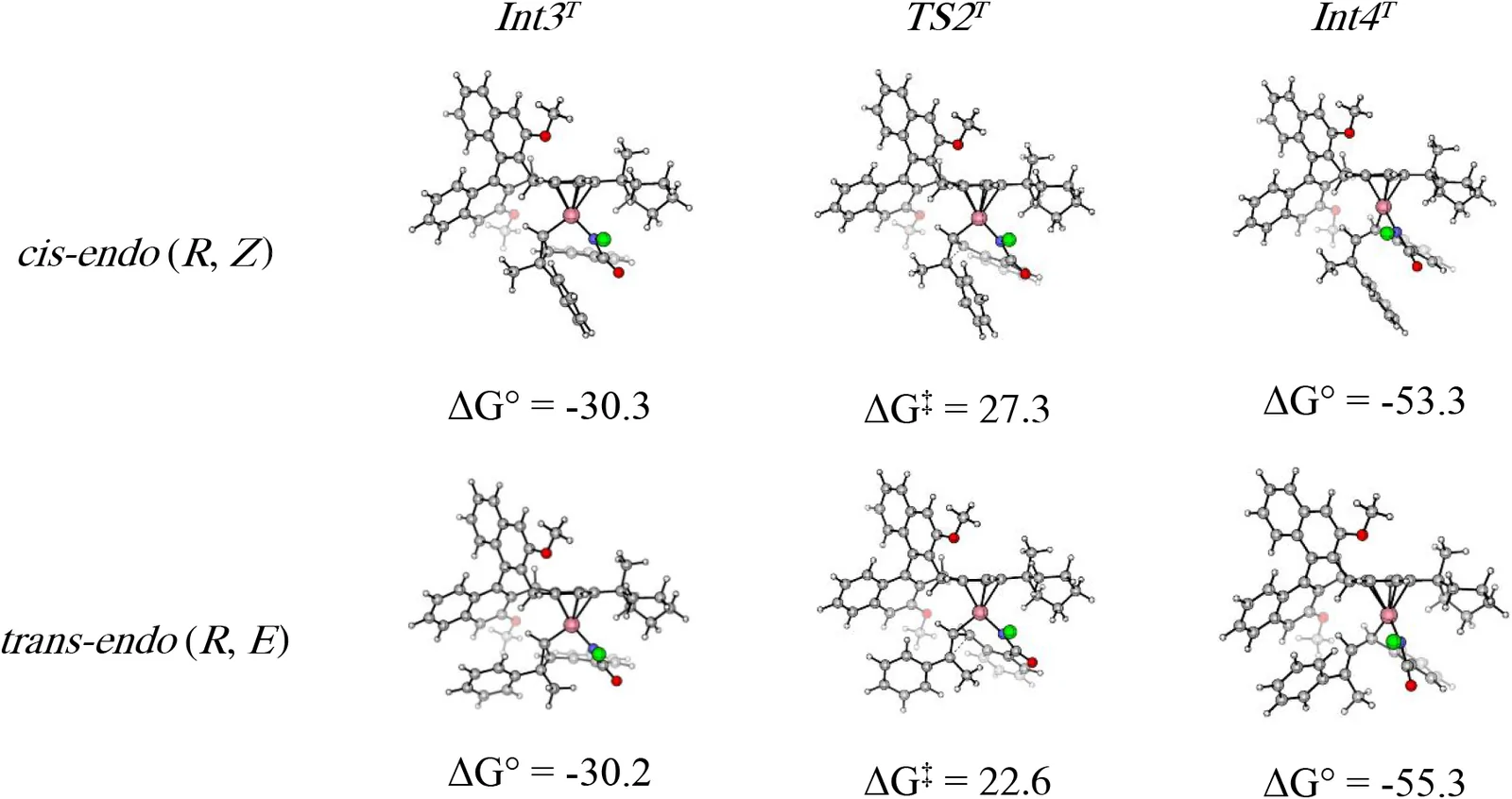
Optimized intermediates and respective transition states in the triplet state, ΔG° and ΔG^‡^ for the relevant reactions in kcal mol^–1^.

ISC is usually a slow process; spin-orbit coupling in transition metals facilitates this process.^21^ The rate of ISC depends on the energy gap between two states (E_f_ – E_i_).^22^ When the singlet and triplet states are close in energy, a greater number of vibrational states can facilitate the transition. Considering this, the energy gap between the singlet and triplet states of **Int4** is smaller (ΔG° = 7.9 and 9.9 kcal mol^–1^ for *cis*-*endo* (*R*, *Z*) and *trans*-*endo* (*R*, *E*), respectively) than that of **Int3** (ΔG° = 13 and 11.3 kcal mol^–1^ for *cis*-*endo* (*R*, *Z*) and *trans*-*endo* (*R*, *E*), respectively). These results are supported by calculations showing that similar CpCo systems relax to the triplet state via a minimum energy crossing point between the singlet and triplet surfaces,^23^ our findings support the conclusion that the ISC occurs more rapidly in **Int4** than in **Int3**.

To confirm mechanistically in which step the ISC occurs, we examined the formation of **Int5**, which is formed from **Int3** ^**T**^ via **TS3** ^**T**^. **Int5** is a six-membered ring intermediate, which is formed experimentally^9^ with Cp^X^Rh­(III) in the singlet state, but not with Cp^X^Co­(III) catalyst. Based on the DFT calculations for this pathway for the *trans-endo* (*R*, *E*) diastereomer, the transition state is 21.4 kcal mol^–1^, and the reaction is highly exergonic (ΔG° = −70.0 kcal mol^–1^). Theoretically, if **Int3** is a triplet, **Int5** is expected to lead to the major product, since it has the lowest transition-state barrier among all the triplet-state pathways. However, since this product is not observed experimentally, the triplet route from **Int3** is ruled out. Given that spin-state changes might arise along the reaction coordinate and affect the overall mechanism,^24^ our results support the conclusion that, similarly to Cramer,^9^ ISC readily occurs in **Int4** rather than in **Int3**. The last step leading to the final product (**Int6**) is highly exergonic (ΔG° = −47.0 kcal mol^–1^) for the *trans*-*endo* (*R*, *E*) product with a TS barrier (**TS4** ^**T**^) of 20.5 kcal mol^–1^.

## Conclusions

In summary, we present the first reported chiral DFT mechanism for the [4+1] annulation of benzamide with cyclopropene, extending Cramer’s previously proposed achiral pathway to a chiral system catalyzed by Cp^X^Co­(III). The mechanism suggested in this manuscript is in line with Cramer’s^9^ mechanism for nonchiral Cp*Co­(III), however, to the best of our knowledge, this work represents the first theoretical study on a reaction mechanism catalyzed by chiral Cp^X^Co. This study demonstrates how different approaches of the substrate, cyclopropene, toward the catalyst lead to distinct diastereoselectivities in the final product. Our calculations indicate that the microkinetic model failed to predict the *trans-endo/cis-endo* product ratio, but the deviation is within the typical intrinsic error margins of DFT calculations. The formation of *exo* products can be excluded due to their significantly higher transition-state barriers. NCI calculations for **TS1** ^**S**^ explain the preference of *endo* over *exo* products. Additionally, analysis of the singlet-triplet energy gaps for **Int3** and **Int4** shows that the cyclopropane ring-opening step takes place at the singlet state, and then, an ISC occurs to form the triplet intermediate **Int4** ^**T**^. Our computational results support this conclusion and are consistent with the experimental absence of the triplet-state by-product (**Int5**). Given the growing interest in chiral Cp^X^M­(III)-catalyzed C–H activation reactions, our findings provide a timely contribution to the mechanistic understanding of chiral Co-catalyzed enantioselective reactions, offering a valuable complement to the mechanistic rationale previously proposed by Cramer and coworkers and might stimulate the discovery of new asymmetric reactions.

## Supplementary Material



## Notes

Scheme S1: reaction profile in DCE and TFE, energies of intermediates and TSs, and XYZ coordinates (PDF)
