# Supplementary material for: Enantioselective [4+1] Annulation of Benzamide with Cyclopropene via Chiral CpXCo(III) Catalysis: A DFT Perspective
Source: ACS Omega. 2026 Sep 10;11(37):55012–9. doi: 10.1021/acsomega.6c01239 (PMC13618818; doi:10.1021/acsomega.6c01239)
Supplement: Supplementary file 1 [file ao6c01239_si_001.pdf]

Enantioselective [4+1] Annulation of Benzamide with Cyclopropene via Chiral $Cp^XCo(III)$ Catalysis: A DFT Perspective

Bat-El Oded, Haya Kornweitz*

Supporting Information

Table of Contents

Scheme S1 – Reaction profile in DCE	S2
Scheme S2 – Reaction profile in TFE	S3
Table S1 – Energies of intermediates and TSs	S3
Figure S1 – 3D optimized structures in singlet state	S5
XYZ Coordinates	S5

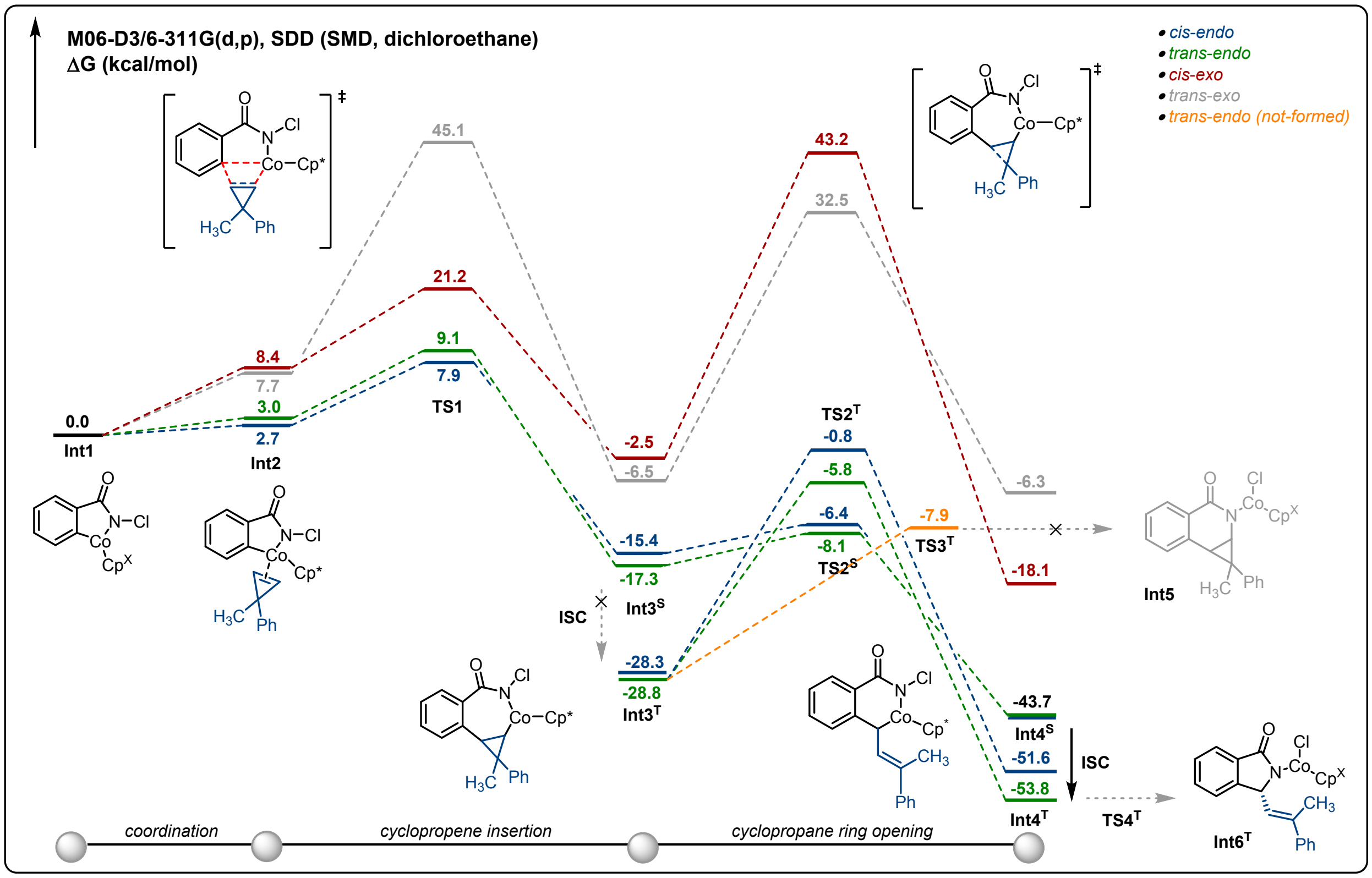

Scheme S1. Energy profile of the reaction pathways. DFT calculations were performed at the M06/SDD-6-311G** /SMD (DCE) level of theory (blue: *cis-endo*, green: *trans-endo*, brown: *cis-exo*, grey: *trans-exo*, orange: not-formed, free energies in kcal mol^{-1}).

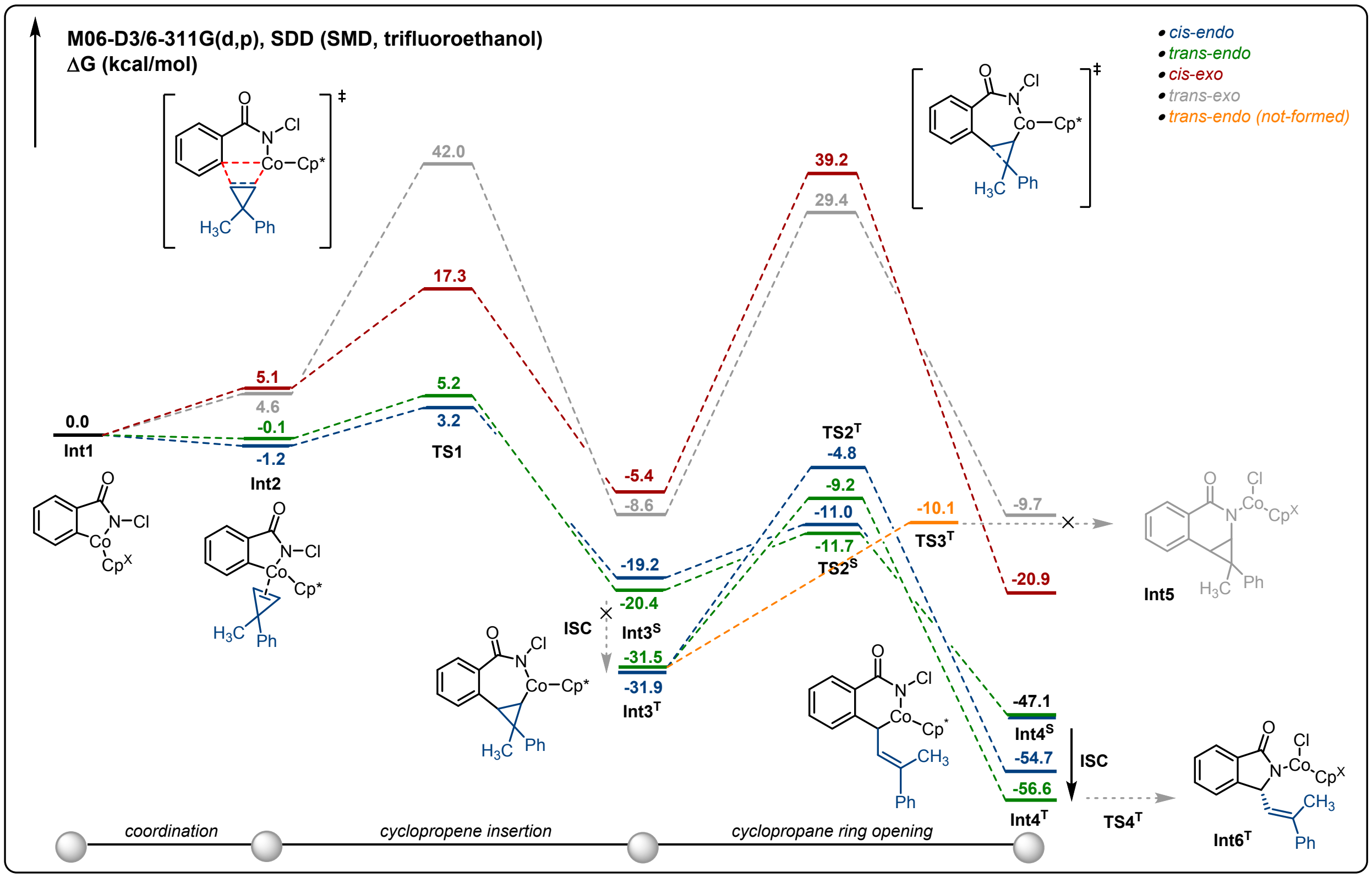

Scheme S2. Energy profile of the reaction pathways. DFT calculations were performed at the M06/SDD-6-311G** /SMD (TFE) level of theory (blue: *cis-endo*, green: *trans-endo*, brown: *cis-exo*, grey: *trans-exo*, orange: not-formed, free energies in kcal mol^{-1}).

Table S1. Computed single point energies in DCE and TFE, corrections (each in Hartree) and number of imaginary frequencies for relevant species.

	Electronic Energy in DCE	Electronic Energy in TFE	Average Electronic Energy	Free Energy Correction	# of imaginary frequency (# cm^{-1})
**Int1^{S}**	-2547.649268	-2547.639969	-2,547.644618	0.636437	0
**Cyclopropene**	-386.7927075	-386.7886025	-386.790655	0.1303	0
**Int2^{S}** – *cis-endo*	-2934.467564	-2934.460568	-2,934.464066	0.796713	0
**Int2^{S}** – *trans-endo*	-2934.466357	-2934.457996	-2,934.462176	0.795908	0
**Int2^{S}** – *cis-exo*	-2934.458272	-2934.450168	-2,934.45422	0.796455	0
**Int2^{S}** – *trans-exo*	-2934.458793	-2934.450324	-2,934.454558	0.795916	0
**TS1^{S}** – *cis-endo*	-2934.460407	-2934.454405	-2,934.457406	0.797807	1 (-285.1)
**TS1^{S}** – *trans-endo*	-2934.458187	-2934.450955	-2,934.454571	0.797472	1 (-291.85)
**TS1^{S}** – *cis-exo*	-2934.43794	-2934.430721	-2,934.434330	0.796573	1 (-176.0)
**TS1^{S}** – *trans-exo*	-2934.402455	-2934.393956	-2,934.398205	0.799188	1 (-224.0)
**Int3^{S}** – *cis-endo*	-2934.499218	-2934.49189	-2,934.495554	0.799379	0

**Int3** ^{S} – *trans-endo*	-2934.501529	-2934.493047	-2,934.497288	0.798669	0
**Int3** ^{S} – *cis-exo*	-2934.479142	-2934.470449	-2,934.474795	0.799891	0
**Int3** ^{S} – *trans-exo*	-2934.487067	-2934.477056	-2,934.482062	0.801397	0
**TS2** ^{S} – *cis-endo*	-2934.484279	-2934.478218	-2,934.481249	0.798802	1 (-201.5)
**TS2** ^{S} – *trans-endo*	-2934.487268	-2934.4797	-2,934.483484	0.799108	1 (-234.0)
**TS2** ^{S} – *cis-exo*	-2934.400581	-2934.393439	-2,934.397010	0.794228	1 (-231.9)
**TS2** ^{S} – *trans-exo*	-2934.418196	-2934.409861	-2,934.414029	0.794884	1 (-172.6)
**Int4** ^{S} – *cis-endo*	-2934.544858	-2934.536992	-2,934.540925	0.799934	0
**Int4** ^{S} – *trans-endo*	-2934.544525	-2934.536416	-2,934.540471	0.799534	0
**Int4** ^{S} – *cis-exo*	-2934.499813	-2934.490835	-2,934.495324	0.795596	0
**Int4** ^{S} – *trans-exo*	-2934.483099	-2934.475068	-2,934.479084	0.797663	0
**Int3** ^{T} – *cis-endo*	-2934.514686	-2934.506913	-2,934.510800	0.794137	0
**Int3** ^{T} – *trans-endo*	-2934.515952	-2934.506804	-2,934.511378	0.794712	0
**TS2** ^{T} – *cis-endo*	-2934.469154	-2934.462066	-2,934.465610	0.792519	1 (-334.8)
**TS2** ^{T} – *trans-endo*	-2934.47842	-2934.470374	-2,934.474397	0.793857	1 (-333.5)
**Int4** ^{T} – *cis-endo*	-2934.54819	-2934.539608	-2,934.543899	0.790474	0
**Int4** ^{T} – *trans-endo*	-2934.554106	-2934.545184	-2,934.549645	0.792961	0
**TS3** ^{T} – *trans-endo*	-2934.478982	-2934.468243	-2,934.473613	0.791097	1 (-374.9)
**Int5** ^{T} – *trans-endo*	-2934.628153	-2934.62079	-2,934.624472	0.796154	0
**TS4** ^{T} – *trans-endo*	-2934.514518	-2934.505769	-2,934.510144	0.786176	1 (-630.9)
**Int6** ^{T} – *trans-endo*	-2934.629285	-2934.621258	-2,934.625272	0.793617	0

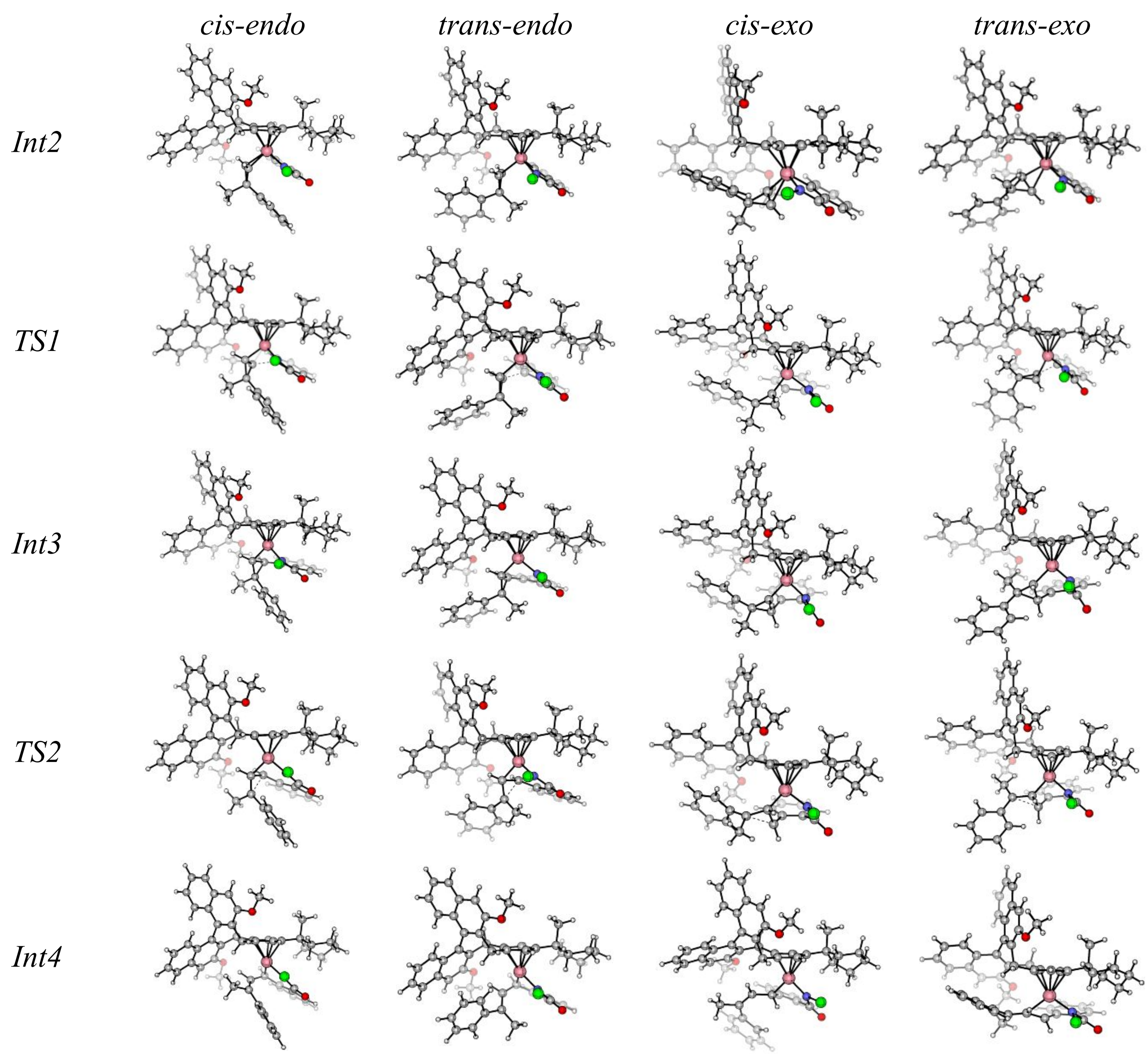

Figure S1. Optimized intermediates and respective transition states in the Singlet state

XYZ Coordinates - zip

https://drive.google.com/drive/folders/1ZuH2nxgYsaCK4hY6UYrn4JNJ-PK2xCvh?usp=sharing
